# Anatomy, ultrastructure and histology of the olfactory organ of the largemouth bass *Micropterus salmoides*, Centrarchidae

**DOI:** 10.1186/s42649-019-0023-3

**Published:** 2019-12-23

**Authors:** Hyun Tae Kim, Seung Woon Yun, Jong Young Park

**Affiliations:** 1 Faculty of Biological Science and Institute for Biodiversity, College of Natural Science, Jeonbuk National University, Jeonju, South Korea; 2 Department of Biological Sciences, College of Natural Sciences, and Institute for Biodiversity Research, Jeonbuk National University, Jeonju, 561-756 South Korea

**Keywords:** The olfactory organ, Rosette structure, Lamellae, Mucous cells, The largemouth bass

## Abstract

The detailed anatomy, ultrastructure and histology of the olfactory organ of *Micropterus salmoides* were investigated by a stereo microscope, a light microscope, and a scanning electron microscope. Its external structure shows a tube-like anterior nostril to stick out and a posterior nostril flat to the skin surface. Meanwhile, its internal structure, the olfactory chamber, contains a fan-shaped rosette structure with 9 to 11 lamellae in adult fish over 35 cm in standard length (SL) and two accessory nasal sacs (ethmoidal and lacrimal sacs) were found. Interestingly, the rosette in young fish under 15 cm in SL was a longitudinal structure in parallel with each of 4–5 lamellae. Histologically, the sensory epithelium (SE) on the olfactory chamber consists of 5 types of cells: olfactory receptor neurons, supporting cells, basal cells, lymphatic cells and mucous cells. In contrast, the non-sensory epithelium (NSE) has stratified epithelial cells, lymphatic cells and mucous cells. The mucous cells of the SE are abundant and distributed densely in one row on the outermost superficial surface, but the one of the NSE are less than the SE. From these results, the olfactory characters of *M. salmoides* may be related with its ecological habit spending in the middle layer of stagnant water contaminated, more or less.

## Introduction

Sensory system, as a process of which teleost fishes respond to diverse stimuli in aquatic milieu, largely is classified into mechano- and chemo- receptions by their differences in a route of innervation, a passage of signal transduction cascade, and a type of stimulation (Hara 1986; Okada 2015). Among them, the olfaction is one of chemoreception that senses only a mixed aquatic compound such as hazard molecules, hormones, bile acid and amino acids dissolved in animal’s habitat, through the dendrites of olfactory receptor neurons (ORNs) to contact the surface of the epithelia (Hara 1994; Hanson et al. 1998). In particular, the signaling of olfaction commences in contact with the ORNs over lamellae of the olfactory chamber and with inflowing water with odors (Hara 1986; Kasumyan 2004). So, the anatomy and histology of the olfactory organ seems to be important in fishes, which are affected by the olfaction, swimming pattern, water volume, bottom structure and water turbidity in habitats (Ferrando et al. 2016; Kim et al. 2016). Sometimes, the olfactory organ has been regarded the archetype for the morphological adaptation to fish-owned lifestyle (Kim and Park 2016; Malick et al. 2018).

The largemouth bass, *Micropterus salmoides*, belonging to the family Centrarchidae is a carnivorous species that shows multi colors with an olive-green to greenish gray, black, a jagged horizontal stripe along each flank in its body according to the variety of habitat where they have adapted, and inhabits lakes, ponds, swamps, brackish water and almost rivers (Kim and Park 2002). The fish is known for an invasive species which has been introduced into the world due to the popularity of its sport fishing (Richardson-Heft et al. 2000). In South Korea, it also has spread widely and rapidly throughout the water system of the Korean Peninsula since it was induced in 1973 for the purpose of fishery resources augmentation (Kim and Park 2002). So far, the colonization of *M. salmoides* in the Korean aquatic environment causes decline or extremely extinction of native freshwater fishes and aquatic insects by predation and competition between them (Lee et al. 2013), so that this is making same serious issues in other countries after its introduction (Pereira and Vitule 2019). With this ecological disturbance of *M. salmoides*, their sensory ability for catching prey or swimming have been tested in previous studies of a foraging success at different light intensities (McMahon and Holanov 1995), the feeding modalities to sensory deprivation (Gardiner and Motta 2012), its color vision (Mitchem et al. 2018), the feeding behavior by colocalization of teeth and taste buds (Linser et al. 1998), in reduction or elimination of its population. Although many teleost fishes rely on a using of vision or olfaction in feeding mechanism (Hara 1986), a valuable data of the olfactory organ conducting olfaction is not revealed in anatomy and histology. So, the aim of this study is to describe anatomy, ultrastructure and histology of the olfactory organ of *M. salmoides* and analyze a relation to its ecology.

## Materials and methods

### Sample preparation

**Fig. 1:**
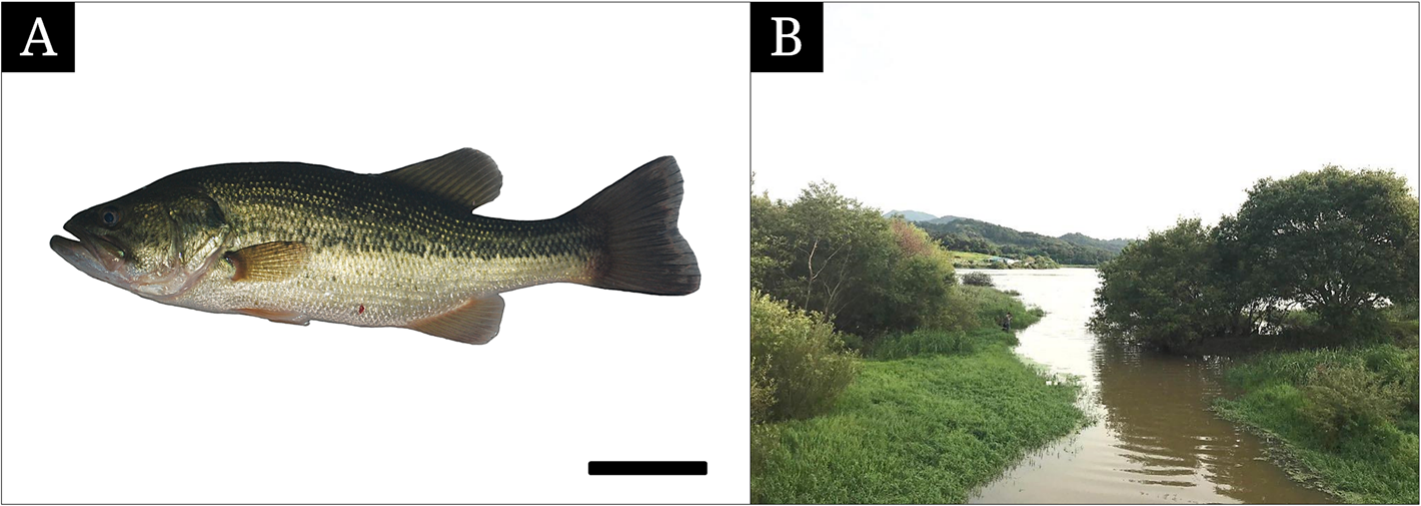
The species photograph **a** and the habitat (**b**, the Daeyul reservoir) of *Micropterus salmoides*. The bar indicates 5 cm

Twenty *M. salmoides* (Fig. 1a) with a variety of sizes (12.5 to 44.2 cm in standard length) were caught using a fishing rod and a cast net (7 × 7 in mesh) from March to October 2019 in the Daeyul reservoir (Fig. 1b, 35°47′36″N, 127°02′25″E) and the Jeonju Stream (35°50′39″N, 127°06′18″E) in South Korea. All procedures for this study were performed according to the rules under the Chonbuk National University Institutional Animal Care and Use Committee. The collected fishes were anesthetized with 0.1% m-aminobenzoic acid ethylester methanesulfonate (MS222, Sigma, St Louis, MO) in the field. Then ten were fixed with 10% neutral formalin solution (pH 7.4) and the rest were kept in 2.5% glutaraldehyde solution (pH 7.4) with 0.1 M phosphate buffer (GA solution), respectively.

### Microscopic investigation

For checking the anatomical structure of the olfactory organ, the five specimens in 10% formalin solution were dissected using an anatomical blade (Feather Safety Razor Co., Ltd., Japan) from the snout of the fish’s head under a stereo microscope (SM; Stemi DV4; Carl Zeiss, Germany). It was filmed for detailed anatomy by a digital camera (TG-3, Olympus, Tokyo, Japan). For light microscopy, the olfactory tissue extracted from the snout were dehydrated properly with a series of graded ethanol solutions, cleaned with xylene, and then embedded in paraffin wax (Paraplast, Oxford) for 24 h. Five-micrometer serial sections of the tissue’s paraffin block were cut by a rotary microtome (Leica 820, Leica Microsystems, Wetzlar, Germany), deparaffinized with xylene, dehydrated in descending series of alcohol solutions. They were stained with Hematoxylin-Eosin (H-E) (Gurr 1956) for a general histology and then observed under a light microscope (LM; Imager A1, Carl Zeiss, Germany). For scanning electron microscopy, the olfactory organ fixed was fixed with GA solution again for 24 h due to the lack of infiltration capacity to the tissue at the first fixation with GA solution. And then it was post-fixed 1% osmium tetroxide (OsO_4_) with 0.1 M phosphate buffer, dehydrated in ascending series of alcohol solutions, transferred to tert-butyl alcohol, freeze-dried in vacuum chamber by a freeze dryer (VFD-21S t-butanol freeze dryer, Shinkuu, Mito, Japan), ion-sputter coated with osmium. They were observed under a scanning electron microscope (SEM; Carl Zeiss, SUPRA 40VP, Germany).

## Results

### Anatomy

**Fig. 2:**
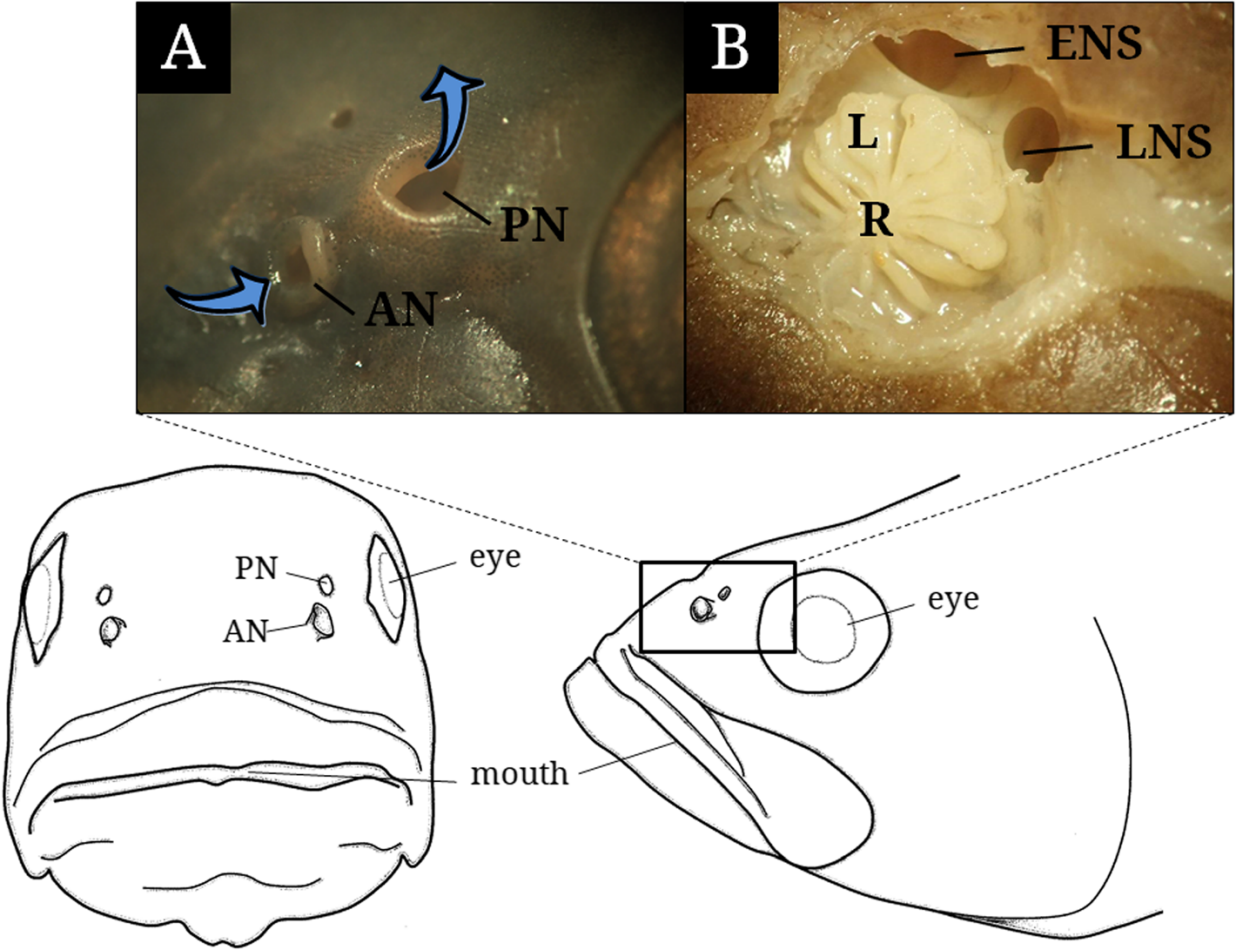
The diagrams in front (left) and side (right) views of the head of *Micropterus salmoides* (adult) and the anatomical structure (external, **a**; internal, **b**) of its olfactory organ. AN, anterior nostril; ENS, ethmoidal accessory nasal sac; L, lamellae; LNS, lacrymal accessory nasal sac; PN, posterior nostril; R, medium raphe

The paired olfactory organs below the eyes of *M. salmoides* consist externally of an anterior nostril and a posterior nostril (Fig. 2). The anterior nostril was tubular structure with a circular opening protruded over the skin and the posterior nostril has an angular opening flat to the skin (Fig. 2a). In the internal view of adult fish over 35 cm in SL, it was confirmed that the olfactory chamber has a rosette structure with 9 to 11 lamellae and two inward holes, ethmoidal and lacrimal accessory nasal sac. The rosette structure appears to be a fan shape with lamella and a medium raphe in arrangement. The distal part of each lamella tends to be larger than the proximal part in its thickness (Fig. 2b).

### Histology

**Fig. 3:**
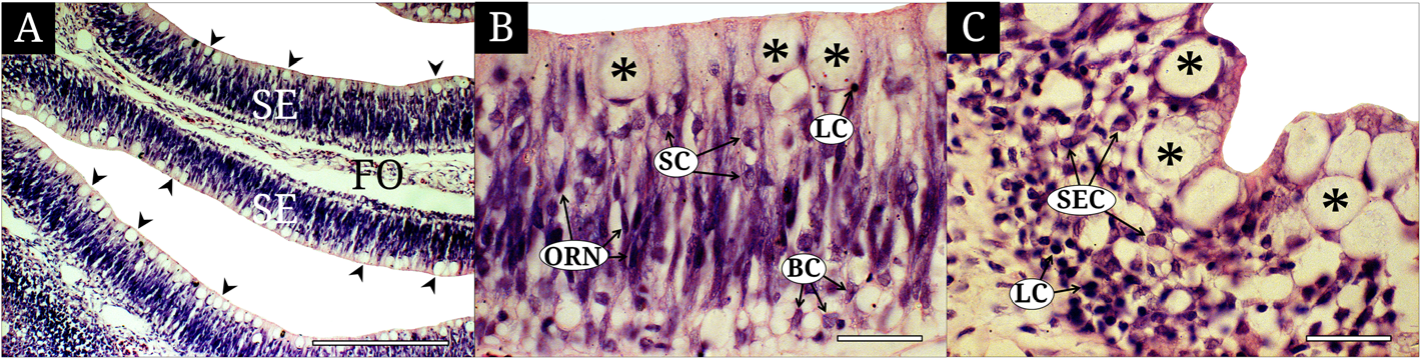
The histological and cytological characteristics of the olfactory epithelium of *Micropterus salmoides*, stained with hematoxylin and eosin. **a** the lamellae with the sensory epithelia on its both sides; **b** the sensory epithelium with olfactory receptor neurons, supporting cells, basal cells, lymphatic cells and mucous cells; **c** the non-sensory epithelium with stratified epithelial cells, lymphatic cells and mucous cells. Arrowhead, mucous cell; asterisk, mucous cell; BC, basal cell; FO, fila olfactoria; LC, lymphatic cell; NSE, non-sensory epithelium; ORN, olfactory receptor neuron; SC, supporting cell; SE, sensory epithelium. The bars indicate 200 μm in A and 20 μm in B and C, respectively

Each olfactory lamella has the sensory epithelium (SE), the non-sensory epithelium (NSE) and the connective tissue, so called fila olfactoria (Fig. 3a). The SE is a pseudostratified layer with variable cells such as the ORNs, supporting cells (SCs), basal cells (BCs), lymphatic cells (LCs) and mucous cells (MCs) (Fig. 3b). With dendrites and axons, the ORNs are a bipolar cell that expands their body to the outermost surface and the basement membrane. They show an elongated nucleus with a deep violet color stained with hematoxylin. The SCs have an elliptical nucleus with weaker violet color than that of the ORNs. Their cytoplasm is cylindrical, being expanded from the bottom to apical surface of the olfactory epithelium. The BCs are a polygonal cell located horizontally and vertically at the basal part of the olfactory epithelium and also show a weak violet color like the SCs. The LCs are a small circular cell with deep dark color stained with hematoxylin and has a very little cytoplasm. The MCs are a large and guttiform cell that is situated at the apical part of the olfactory epithelium and appears abundantly on the SE. They have a flat nucleus with a deep violet color at the bottom of the cell body (Fig. 3b).

Meanwhile, the NSE is a stratified squamous epithelium that shows an irregularly curved line in its surface (Fig. 3c). It consists of stratified epithelial cells (SECs), the LCs and the MCs. The SECs are a polygonal cell with nucleus located randomly in its wide cytoplasm and a weak violet color. Sharing the same characters to that of the SE, the LCs are visible in all part of the epithelial layer, and numerous in number. The MCs are dense and continuous along the surface line of the NSE and show a faint color negative for hematoxylin in their cytoplasm. Their cytological characteristics are similar to that of the SE (Fig. 3c).

**Fig. 4:**
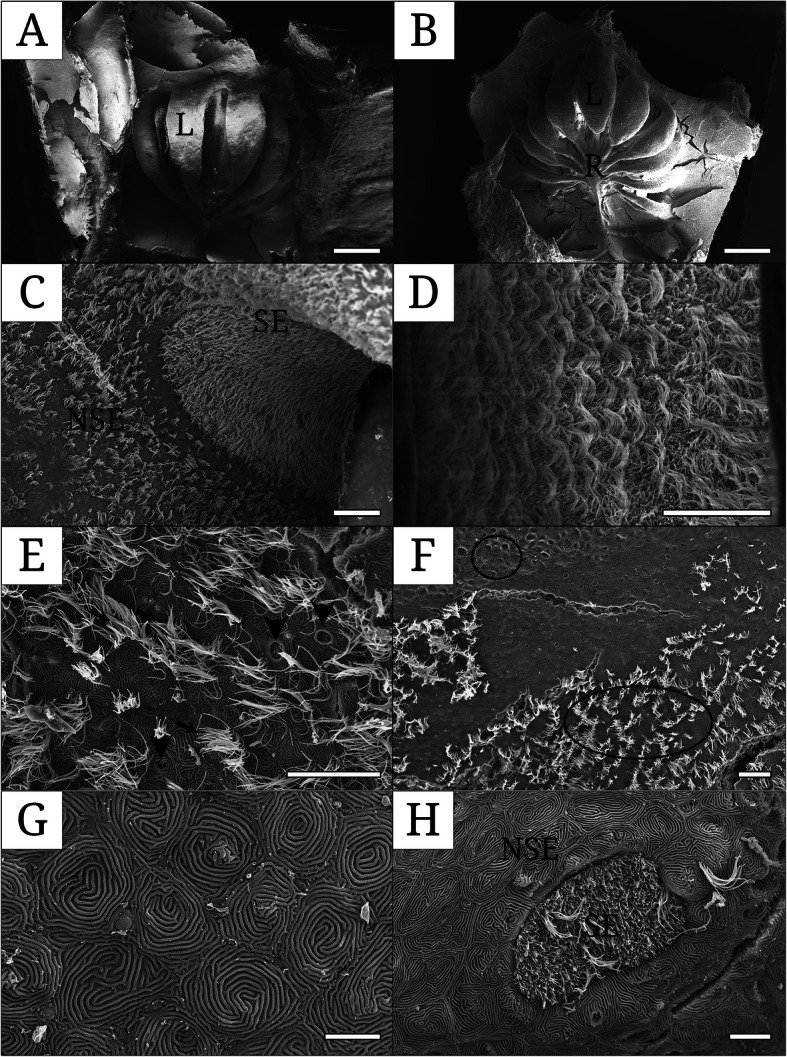
Scanning electron micrographs showing the olfactory lamellar surface of *Micropterus salmoides*. **a** the olfactory rosette of young fish under 15 cm (SL); **b** the olfactory rosette of adult fish over 35 cm (SL); **c** the surface area divided into two regions, sensory and non-sensory epitheliums; **d** the sensory epithelium with numerous cilia; **e** the non-sensory epithelium with mucous openings and motile cilia; **f** the non-sensory epithelium consisting of two regions, with and without cilia; **g** the non-sensory epithelium with spiral patterns of microridges; **h** the sensory epithelia among the non-sensory epithelium. Arrow, cilia; arrowhead, mucous opening; circle, non-sensory area without cilia; ellipse, non-sensory area with cilia; L, lamellae; R, medium raphe; SE, sensory epithelium; NSE, non-sensory epithelium. The bars indicate 200 μm in A, 500 μm in B, 20 μm in C, D, E, F, 5 μm in G and H, respectively

The rosette structure of *M. salmoides* transforms a longitudinal type in parallel with one another (Fig. 4a) into a fan-shaped type arranged radially on the basis of the medium raphe (Fig. 4b). The former type appears in young fish under 15 cm. As the young fish grow, adult fish over 35 cm change to the latter, which is over twice the diameter size of the former. The SE area on the olfactory lamella shows continuous distribution in its developmental pattern on the surface (Fig. 4d) with numerous and dense cilia. In contrast, the NSE shows two distinct parts: A type of the region mixed with ciliated and non-ciliated cells (Fig. 4e), and B type of the only non-ciliated region to form spiral pattern of microridges (Fig. 4g). The NSE in the outer margin of the lamella and the connected part of the inner floor and the lamella shows A type (Fig. 4c) whereas in the medium raphe and the inner floor it is B type (Fig. 4f). Rarely, the patches of the SE are observable among A type of the NSE (Fig. 4h).

## Discussion

The family Centrarchidae prefers to live around aquatic vegetation and hide near it in warm and slower-moving water (Berra 2001). Among them, although *M. salmoides* depends on its well-developed eyes to catch prey in sight (Crowl 1989), it is clear that other sensory organs are needed. In particular, olfaction is very useful to perceive any change in aquatic environments that they may be faced (Janzow 1978; Kubitza et al. 1997).

The anatomical features of *M. salmoides* are as follows: a tubular-anterior nostril to stick out, fan-shaped lamellae with 9 to 11 units arranged radially on the medium raphe, and two accessory nasal sacs. The tubular-anterior nostril has been well-known in most of the Perciformes as a tube- or a tentacle-like hole located at a differing position of their snout (Murase 2007; Padate et al. 2017; Zeiske et al. 1992). Generally, such nostril has been identified in benthic or settled fishes, to maintain a benthic strategy, move slowly and remain motionless in at least part of their whole life history (Kasumyan 2004; Cox 2008). *M. salmoides,* however, shows meso-pelagic life to swim middle or upper layer of the water (Kim and Park 2002). Despite the difference in their ecological aspects, it is very interesting in that *M. salmoides* has a tubular anterior nostril. The function of two accessory nasal sacs seen in this fish has been known as an intentional water ventilation of the olfactory chamber in habitat environment with standing or slow water (Pashchenko and Kasumyan 2019).

The rosette structure, a lamellar organization projected from the inner wall of the olfactory chamber, has been reported in adult of many teleost fishes (Hara 1986; Atta 2013). In the arrangement of rosette with lamellae, Yamamoto (1982) classified into eight types: i) absence of lamella, ii) longitudinal one, iii) transverse one, iv) longitudinal lamellae parallel to another units, v) fan-shaped lamellae, vi) lamellae radiating from a raphe, vii) lamellae arranged transversely or obliquely to an elongated raphe, viii) transverse lamellae with mostly same size arranged from a long raphe. With above types, Kasumyan (2004) added two cases: i) two longitudinal lamellae, ii) lamellae of same size radiating from centered raphe. The above reports confirmed that the rosette structure remain unchanged for their whole life time. Unlike the reports, the rosette structure of *M. salmoides* shows the changes by growth: longitudinal lamellae in younger fish transforms into fan-shaped lamellae in adult fish. There were only some studies that adult fish tends to have a larger number of lamellae than young fish to increase the area in contact with odor and the ORNs as the fish’s olfactory sensitivity may be related to increase in lamellae number (Kasumyan 2004; Kudo et al. 2009). The general anatomy of the olfactory organ in the same species remains unchanged regardless size and age of fishes, and habitat they live in (Zeiske et al. 1992; Kasumyan 2004). However, the arrangement of the rosette structure is different and unique by species (Yamamoto 1982; Ferrando et al. 2017). Meanwhile, the lamellae number is variable between individuals with growth, size, and age (Tilney and Hecht 1990).

The morphology of the lamellae also is known to be affected by a ventilatory aspect of water in the olfactory chamber and fish’s swimming (Ferrando et al. 2016). Consequently, such transformation for *M. salmoides*, is a rare phenomenon in the olfactory organ of teleost fishes.

The olfactory epithelium of teleost fishes has generally the ORNs, the SCs, the BC within the SE, the SECs and the MCs within the NSE (Hara 1994). Sometimes, very few MCs occur on the SE (Hara 1986). Unusually, *M. salmoides* is remarkable that the MCs are lined densely and abundantly in the superficial surface of the SE. The presence of the MC in the SE may be a unique phenomenon. The MCs produce more mucus when fish face a habitat environment with contaminated water chemically (Kasumyan 2004). Therefore, as *M. salmoides* has a strong tolerance in turbid or contaminated water, the appearance of the MCs in the SE may be related with the hypothesis reported above regarding at least its ecological aspects.

Numerous cilia of the SE and many LCs of the NSE in *M. salmoides* are found. According to Doving (1977), it was studied the cilia assist the movement of water or mucus over the epithelia. The LCs are functioned as part of cell immunity (Lieschke and Trede 2009).

From the above data, the gross structure and histological characteristics of the olfactory organ of *M. salmoides* in mesopelagic ecology may be likely considered as one of strategy to adapt for stagnant and more contaminated water rather than clear and rapid one.

## Conclusion

The paired olfactory organ of *Micropterus salmoides* shows remarkable characters: i) a tubular anterior nostril to stick out, ii) a posterior nostril flat to the skin surface, iii) two accessory nasal sacs, ethmoidal and lacrimal sacs, v) a rosette structure transforming longitudinal lamellae (adult fish) in parallel with each other into a fan-shaped lamellae (younger fish) in arrangement, vi) mucous cells being dense and continuous on the sensory epithelium, vii) numerous lymphatic cells in the non-sensory epithelium. Such olfactory characters may be related with a habit spending in the middle layer of stagnant water contaminated, more or less.

## Data Availability

Not applicable.

## Abbreviations

BC: basal cell
GA: glutaraldehyde
LC: lymphatic cell
LM: light microscope
MC: mucous cell
NSE: non-sensory epithelium
ORN: olfactory receptor neuron
SC: supporting cell
SE: sensory epithelium
SEC: stratified epithelial cell
SEM: scanning electron microscope
SL: standard length
SM: stereo microscope
